# Placental Hofbauer cells assemble and sequester HIV-1 in tetraspanin and DC SIGN positive compartments that are accessible to neutralizing antibodies

**DOI:** 10.1186/1471-2334-14-S3-O5

**Published:** 2014-05-27

**Authors:** Erica L Johnson, Hin Chu, Siddappa N Byrareddy, Paul Spearman, Rana Chakraborty

**Affiliations:** 1 Department of Pediatrics; Emory University; Atlanta, Georgia, 30322, USA; 2 Children’s Center for Immunology and Vaccines; Children’s Healthcare of Atlanta, USA; 3 Department of Pathology & Laboratory Medicine, Emory University School of Medicine, & Emory Vaccine Center, Atlanta, Georgia, 30322, USA

## Background

Within monocyte derived macrophages, HIV-1 accumulates in virus containing compartments (VCCs) that are largely inaccessible to the external environment, which implicate these cells as HIV-1 reservoirs. During mother to child transmission, placental macrophages (Hofbauer Cells [HCs]) are viral targets, and have shown to be infected *in vivo* and sustain low levels of viral replication *in vitro*, however, the risk of *in utero* transmission is less than 7%. The role of these primary macrophages as viral reservoirs is largely undefined.

## Methods

With consent, term placentas from 20 HIV-1 seronegative women were obtained following caesarian section. VCCs were evaluated with 3D confocal microscopy and correlated with electron microscopy. Co localization R values (Pearson’s correlation) were quantified with co localization module of Volocity5.2.1. Replication kinetics following siRNA and neutralization studies were evaluated using p24 ELISA.

## Results

We demonstrate primary HCs assemble and sequester HIV-1 in VCCs with localization specific to intracellular vesicles and the plasma membrane. These compartments are enriched in endosomal/lysosomal markers, including CD9, CD81, CD63 and LAMP 1, along with DC SIGN. Following internalization, we observed HIV-1 accumulation in acidified compartments. Remarkably, these compartments are accessible via the cell surface and can be targeted by exogenously applied small molecules and HIV-1 specific neutralizing antibodies (NAbs). In addition, broadly NAbs (4E10 and VRC01) neutralized HIV-1 infected HCs and involve the participation of FcγRI for inhibition.

## Conclusion

These findings suggest placental HCs possess intrinsic adaptations facilitating sequestration of HIV-1, and may serve as a protective viral reservoir to permit viral degradation, neutralization and antiretroviral drug entry *in utero*.

